# Allopatric humpback whales of differing generations share call types between foraging and wintering grounds

**DOI:** 10.1038/s41598-021-95601-7

**Published:** 2021-08-11

**Authors:** Mikala V. Epp, Michelle E. H. Fournet, Gregory K. Silber, Gail K. Davoren

**Affiliations:** 1 Department of Biological Sciences, University of Manitoba, Winnipeg, MB R3T 2N2 Canada; 2 K. Lisa Yang Center for Conservation Bioacoustics, Cornell Lab of Ornithology, Cornell Lab of Ornithology, Cornell University, Ithaca, NY USA; 3 Sound Science Research Collective, Juneau, AK USA; 4 Smultea Sciences, Washington Grove, MD USA

**Keywords:** Ecology, Behavioural ecology

## Abstract

Humpback whales (*Megaptera novaeangliae*) are a cosmopolitan baleen whale species with geographically isolated lineages. Despite last sharing an ancestor ~ 2–3 million years ago, Atlantic and Pacific foraging populations share five call types. Whether these call types are also shared between allopatric breeding and foraging populations is unclear, but would provide further evidence that some call types are ubiquitous and fixed. We investigated whether these five call types were present on a contemporary foraging ground (Newfoundland, 2015–2016) and a historic breeding ground (Hawaii, 1981–1982). Calls were classified using aural/visual (AV) characteristics; 16 relevant acoustic variables were measured and a Principal Component Analysis (PCA) was used to examine within-call and between-population variation. To assess whether between-population variation influenced classification, all 16 variables were included in classification and regression tree (CART) and random forest analyses (RF). All five call types were identified in both populations. Between-population variation in combined acoustic variables (PC1, PC2, PC3) was lower within call types than among call types, and high agreement between AV and quantitative classification (CART: 83% agreement; RF: 77% agreement) suggested that acoustic characteristics were more similar within than among call types. Findings indicate that these five call types are shared across allopatric populations, generations, and behavioural contexts.

## Introduction

Many studies have investigated the roles that genetics and learning play in the types of signals used by acoustically communicating animals^1^. The number and type of acoustic signals are sometimes under strong genetic control resulting in innate signals (i.e. calls/signals that are unlearned)^2,3^, while aspects of the repertoire in other species change over time through learning^4–7^, such as vertical cultural transmission (mother to offspring)^8,9^ or horizontal cultural transmission (between adults)^1,10–12^. Some acoustic repertoires, whether learned or innate, change substantially over time^13,14^, while others change minimally over time (i.e. stable) and are detected across generations (i.e. fixed^1,15,16^). Examining plasticity in vocal repertoires can provide insight into the selective pressures acting on vocal behaviour^16,17^, the function of calls^18–20^, and the extent of the ability of a species to respond to environmental change^13,14,21,22^.

Regardless of whether innate or learned, call types that persist in the repertoire over lifetimes and/or generations, and are shared among allopatric groups and contexts, likely serve an important function. In addition, call types of marine mammals that are fixed and ubiquitous would be ideal candidates for global passive acoustic monitoring efforts. Indeed, there is evidence of shared calls, with few differences in characteristics, among allopatric populations of some marine mammals (killer whales (*Orcinus orca*)^23,24^; humpback whales (*Megaptera novaengliae*)^25^; fin whales (*Balaenoptera physalus*)^26^). Additionally, portions of vocal repertoires of a number of marine mammal species appear to be stable over the lifetime of individuals and possibly fixed across generations, such as killer whales^27–29^, harp seals (*Pagophilus groenlandicus*^30,31^), bearded seals (*Erignathus barbatus*^32^), and bottlenose dolphins (*Tursiops truncatus*^15,33^).

Humpback whales are a globally distributed, highly vocal, and migratory species whose social and acoustic behaviour varies throughout their annual migratory cycle. Within each ocean basin, individuals appear to return annually to high latitude foraging grounds^34–36^ and lower latitude reproductive regions, where breeding and calving are thought to occur^37,38^. Exchange among ocean basins (North Atlanic, North Pacific, and Southern Oceans) is extremely rare^39–41^, and the humpback whale groups in these basins are considered to be separate lineages^41^. Social interactions differ between foraging grounds and breeding grounds. With some exceptions, humpback whale social interactions at high latitudes are inconspicuous and primarily related to feeding^42,43^. Aggressive behaviour appears to be rare on foraging grounds^43^, and humpback whales typically feed in groups^43^ or solitarily^19^ and occasionally engage in coordinated group foraging^43,44^. By contrast, feeding has only been sporadically observed on breeding grounds^45^, but physically aggressive surface-active groups of male humpback whales are commonly observed in association with what is assumed to be reproductive behaviors^46–48^.

Humpback whale vocal behaviour also varies throughout the annual cycle. Humpback whale song is produced primarily by males^49–51^ and is mainly associated with reproductive behavior at low latitudes, although song also occurs regularly on foraging grounds^51–53^. Song consists of a rhythmic, repeated, and consistent pattern with units (or individual calls) combining into phrases, then into themes^49–51^. Songs are organized in ‘sessions’ that can continue uninterrupted for hours^49^. By contrast, humpback whale calls (a.k.a. non-song calls or social sounds/vocalizations)^46,54–57^ are shorter, generally un-patterned vocalizations^46,56^ that occur across the humpback whale migratory range and are produced by all age and sex classes^55,58^. While song continually changes, such that different call types and call type combinations are used at different times^17,59,60^, some call types within a single population persist in the acoustic repertoire over decades^57,61^. Call types in the humpback whale vocal repertoire are highly diverse, with over 40 individual calls types identified from populations around the world^54,56,62^. This diverse call repertoire is in sharp contrast to other baleen whale species [e.g., blue whales (*Balaenoptera musculus*), fin whales, minke whales (*Balaenoptera acutorostrata*)] whose call repertoires are limited to very few call types or structures^63–65^.

**Figure 1:**
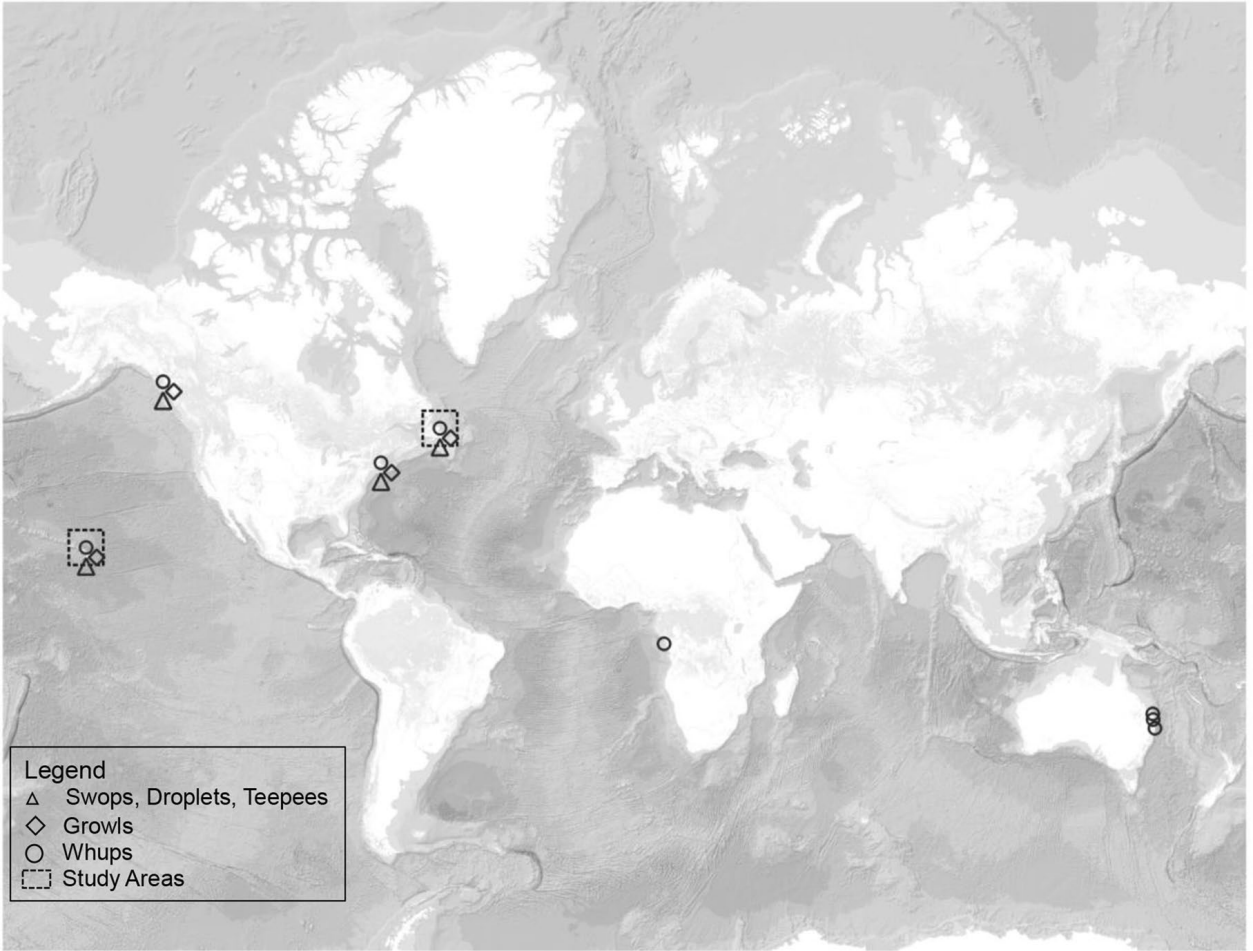
World map showing the locations of the two study areas (Newfoundland^86^ and Hawaii^46^) in boxes, along with other regions where one or more of the five call types have been described, representing two migration routes and two foraging grounds. From left to right on the map (excluding the study sites): Southeast Alaska, USA (foraging) where all five call types were identified and found to be stable over 36 years (1976–2012)^25,54,57^; Massachusetts Bay, USA (foraging; 2008)^25^; Angola, Africa (migration; 2012)^62^; Harvey Bay and Byron Bay (1997, 2003, 2004, 2008), Australia (migration) where whups (called ‘wops’) were stable over 11 years^61^; and other locations on the East Coast of Australia (migration; 2002–2004^56^; 2002–2004, 2008^67^). This map was created using QGIS 3.8.3-Zanzibar (https://qgis.org/en/site/forusers/download.html) using the ESRI Ocean basemap (https://services.arcgisonline.com/ArcGIS/rest/services/Ocean/World_Ocean_Base/MapServer/tile/%7Bz%7D/%7By%7D/%7Bx%7D&zmax=20&zmin=0) accessed on October 21, 2020.

Only two humpback whale call repertoires have been quantitatively compared between populations, to date. Five relatively common and previously described humpback whale call types^54,61^,—“droplets”, “swops”, “teepees”, “growls”, and “whups”^54,56,66^—are shared between humpback whale populations on allopatric foraging grounds in the North Atlantic and North Pacific (Fig. 1)^25^. Moreover, in the North Pacific, the same five call types were detected in the repertoire over a 36-year period, indicating multi-generational peristence^57^. Though not formally compared, qualitatively similar call types were detected during migration in Angola, Africa^62^. The repertoire produced by humpback whales from the east Australian population, migrating past Southeast Queensland, Australia (Fig. 1)^56,61,67^ also contains qualitatively similar call types, several of which were stable over an 11 year period (1997–2008^61^). Qualitatively, these call types appear to be shared across behavioural contexts (i.e., migration and foraging) and non-overlapping regions^25,54,56,61,62^ (Fig. 1), but, to-date, formal, quantitative comparisons of call repertoires across behavioral contexts are lacking.

To examine whether a portion of the humpback whale call repertoire is shared among contexts (foraging and reproduction) and over time, we used aural/visual (AV) characteristics of our recordings to identify whether the five previously described call types were present in a feeding area (in waters off Newfoundland, 2015, 2016) and also in a breeding area (the Hawaiian islands), more than three decades prior (1981–1982^46^). Of the calls present, we first examined between-population variance within each call type and then examined among-call type variance by determining whether quantitative methods would classify the calls present into call types regardless of the population of humpback whales producing them. These five call types were chosen as a template for our analysis as they were previously found to be shared among allopatric foraging grounds^25^ and to be stable^57^. Additionally, these five call types have been proposed to function in maintaining contact or in other close range communication in various regions^55,68,69^, and, thus, are likely to be ubiquitous and important to all humpback populations, regardless of context. Examining our recordings from Newfoundland and Hawaii provided the opportunity to examine calls from geographically isolated populations, two distinct behavioural contexts, as well as across 3–5 generations—given that the age of first parturition in humpback whales is between 10 and 12 years old^70,71^, but as early as five years old in the North Atlantic^72^. If call types are shared by allopatric populations with contrasting behavior across generational time, this would suggest that those call types are fixed and serve an important role in the humpback whale repertoire. This finding may also suggest that these calls form part of a foundation to the global humpback whale call repertoire and may be candidates for global passive acoustic monitoring.

## Results

**Figure 2:**
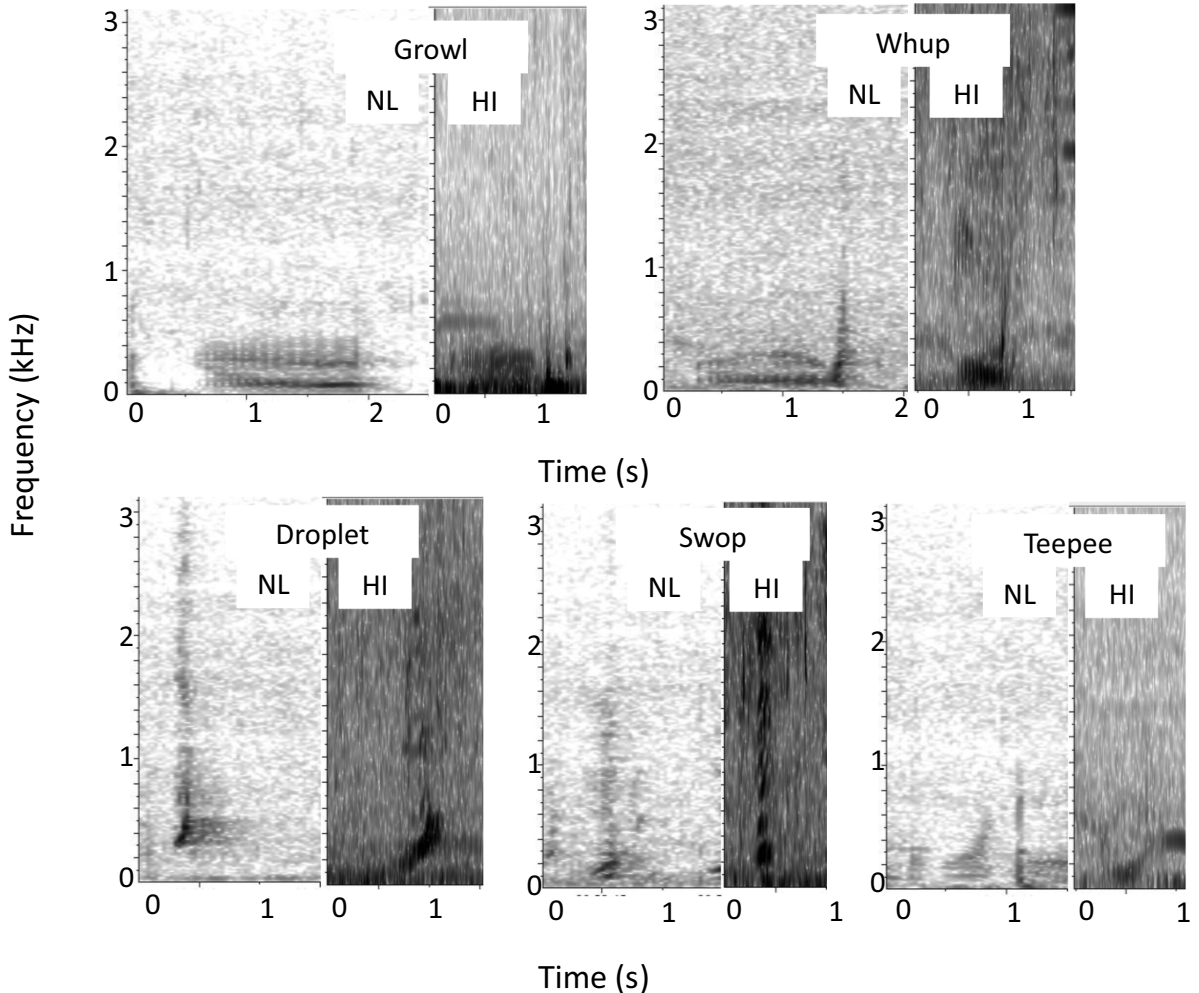
Spectrograms examples (Hann window, 8192 (NL) and 32,768 (HI) Discrete Fourier Transform, 2.93 Hz resolution, and 50% overlap) of each of the five call types from each population, *NL* Newfoundland during 2015, 2016, *HI* Hawaii during 1981, 1982.

In total, ~ 453 h of recordings were reviewed (420 h Newfoundland (NL), 30 h 51 m Hawaii (HI); Table 1). A total of 1841 calls (n = 1369 NL, n = 472 HI) met the inclusion criteria (see “Methods” for details). All five call types (swops, droplets, teepees, growls, whups) were identified in both regions according to aural/visual (AV) classification (Table 2; Fig. 2). The growl call type comprised the largest proportion of calls in NL (37%, n = 502), but the smallest proportion in HI (3%, n = 13; Table 2), while the droplet call type was the most prevalent call in HI (51%, n = 239) and the least prevalent in NL (7%, n = 102; Table 2). Qualitatively, many of the calls in HI had less clear structure and definition on the spectrograms than those in NL (Fig. 2), possibly due to differences in recording equipment and distance to the hydrophone (Table 1). The swop and teepee call types occurred more often in bouts and often in longer bouts in HI (maximum number of calls per bout: swops = 22, teepees = 3) than in NL (swops = 15, teepees = 9; Table 2).

**Table 1:** Description of recording equipment, settings, and contexts for each study area.

Location	Context	Recording years, hours, days	Recording equipment and collection method	Sampling rate
West Maui, Hawaii (~ 21°44′N, 155°55′W)^46,88^	Breeding ground	1981, 1982 30 h 51 m, 23 days	Nakamichi 550 portable cassette recorder with Gould CH-17U, Aquadyne AQ-17 or sonobuoy hydrophones Barcus Berry preamplifier 16 bit Dip hydrophone off of boat—10–16 m depth, when whales were within 200 m of boat Collected by Gregory K. Silber^46,88^	96 kHz
Northeast Newfoundland (49°15′N, 53°26′W)	Foraging ground	2015, 2016 420 h, 18 days	*Wildlife Acoustics* SM2M marine recorder with HTI-96 MIN hydrophone (High Tech Inc., Long Beach, Massachusetts, USA); recording bandwidth: 2 Hz–48 kHz; sensitivity − 165 dB re: 1 V/µPa; flat frequency response from 200 Hz to 10 kHz (Wildlife Acoustics Inc., 2013); 12 dB gain, 3 Hz high pass filter, 16 bit Bottom mounted hydrophone—~ 30 m depth, 3 m off the ocean floor, passive, continuous recording Collected by Mikala V. Epp and Gail K. Davoren	24 kHz

**Table 2:** Mean (SE) for each call type from both regions (Newfoundland—NL, Hawaii—HI) with sample sizes (n).

Call Type	Region	n	Bout	PFC	Lower	Upper	Peak	Band	Median	Range	Trend	Start	End	Dur	Entropy	Ampmod	Freqmod	Upsweep
Droplet	HI	239	1.06 (0.02)	23.67 (0.55)	155.5 (4.29)	546.18 (16.63)	266.27 (5.72)	390.68 (16.16)	283.81 (5.65)	0.31 (0.01)	0.41 (0.01)	181.79 (4.59)	448.01 (6.78)	0.34 (0)	34.57 (1.41)	2.07 (0.08)	2.07 (0.08)	96.61 (0.73)
	NL	102	1.63 (0.09)	1.85 (0.13)	173.11 (7.9)	755.31 (35.82)	269.93 (12.82)	582.2 (35.89)	293.11 (11.18)	0.27 (0.01)	0.6 (0.02)	189.55 (7.48)	356.09 (18.15)	0.24 (0)	43.95 (2.25)	0.2 (0.07)	0.2 (0.07)	91.13 (2.35)
Swop	HI	116	4.34 (0.42)	14.76 (0.56)	183.71 (5.92)	1634.61 (92.11)	420.71 (33.96)	1450.9 (91.44)	583.22 (39.58)	0.15 (0.01)	0.87 (0.02)	229.7 (7.09)	266.29 (6.59)	0.26 (0.01)	88.26 (5.57)	0.6 (0.11)	0.6 (0.11)	48.53 (4.14)
	NL	360	2.14 (0.15)	2.07 (0.07)	108.48 (2.08)	678.7 (16.58)	215.97 (5.38)	570.22 (16.51)	254.57 (4.56)	0.19 (0)	0.91 (0.01)	143.3 (2.56)	170.41 (4.5)	0.25 (0)	42.95 (1.21)	0.22 (0.04)	0.22 (0.04)	80.95 (1.77)
Teepee	HI	66	4.41 (0.48)	18.59 (0.99)	91.4 (6.8)	793.19 (97.17)	269.62 (42.43)	701.79 (94.61)	290.89 (36.13)	0.18 (0.01)	0.96 (0.11)	149.18 (10.96)	172.37 (9.92)	0.29 (0.01)	47.69 (6.19)	0.92 (0.16)	0.92 (0.16)	61.93 (5.12)
	NL	114	2.93 (0.21)	4.41 (0.24)	75.27 (3.34)	474.2 (23.57)	152.14 (7.66)	398.93 (23.29)	180.32 (7.05)	0.18 (0.01)	0.99 (0.01)	102.93 (3.41)	104.57 (3.59)	0.35 (0.01)	28.19 (1.47)	1.71 (0.12)	1.71 (0.12)	76.37 (2.90)
Growl	HI	13	1.08 (0.08)	66.38 (7.15)	118.43 (24.4)	515.51 (156.75)	202.37 (30.93)	397.09 (150.67)	218.4 (32.61)	0.31 (0.07)	0.94 (0.03)	175.02 (19.77)	187.24 (20.96)	0.79 (0.07)	21.34 (4.2)	1.38 (0.11)	1.38 (0.11)	34.92 (7.24)
	NL	502	1.09 (0.01)	12.88 (0.22)	44.95 (1.02)	309.62 (6.35)	120.31 (3.32)	264.68 (6.36)	136.89 (2.87)	0.16 (0)	0.89 (0.01)	66.61 (1.01)	75.38 (1.08)	0.69 (0.01)	17.9 (0.36)	1.67 (0.02)	1.81 (0.03)	45.37 (0.99)
Whup	HI	38	1.47 (0.12)	56.26 (5.48)	59.29 (4.98)	1034.03 (146.33)	249.1 (60.48)	974.74 (147.31)	394.9 (67.31)	0.11 (0.02)	0.84 (0.03)	114.21 (6.3)	136.39 (6.39)	0.71 (0.06)	62.71 (10.64)	1.92 (0.11)	1.86 (0.1)	56.55 (4.49)
	NL	291	1.15 (0.02)	12.25 (0.34)	47.46 (1.27)	374.6 (11.23)	108.27 (3.41)	327.14 (11.09)	132.4 (3.43)	0.15 (0.01)	0.84 (0.01)	68.18 (1.32)	81.13 (1.28)	0.67 (0.02)	21.59 (0.67)	1.76 (0.03)	1.78 (0.04)	51.15 (1.37)

Variable abbreviations correspond to those in Table 4.

**Figure 3:**
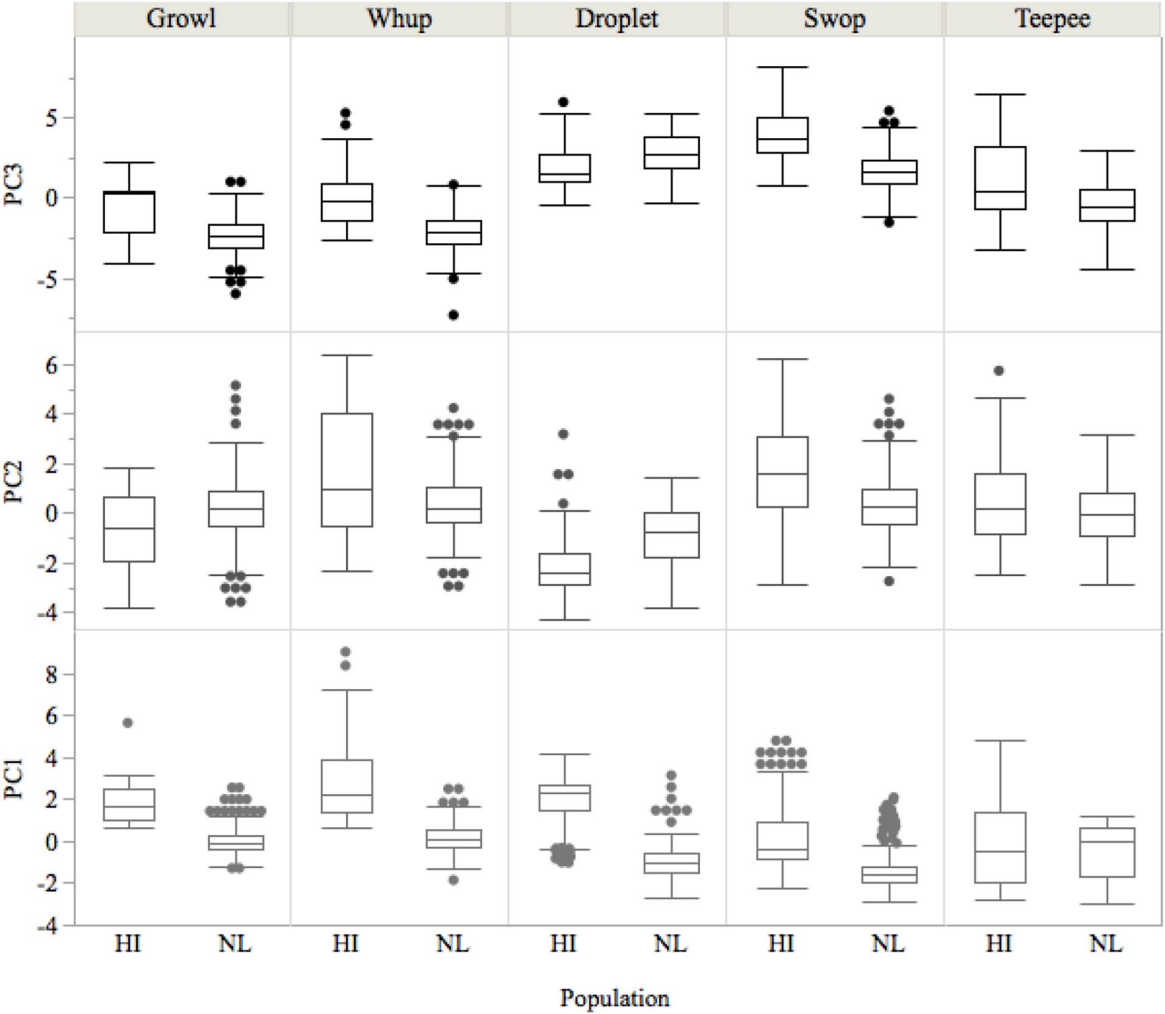
PC1, PC2, PC3 by call type and population showing between-population variation in acoustic parameters within call types as well as variation among call types. Boxplots show the mean (middle horizontal bar), 25th percentile (lower bar), 75th percentile (upper bar), and outliers. Positive PC1 values refer to calls of higher frequency and shorter duration, while negative PC1 values refer to calls of lower frequency and longer duration. Positive PC2 values refer to calls with a larger range in frequency and higher entropy, while more negative PC2 values refer to calls with a smaller frequency range and lower entropy. Positive PC3 values refer to calls with more variation in frequency and amplitude, while more negative PC3 values refer to calls with less amplitude and frequency variation.

A principal components analysis (PCA) revealed that the first three axes had eigenvalues greater than 1 (PC1: 6.4, PC2: 2.6, PC3: 2.1) and accounted for 69.2% of the variance (PC1: 39.7%, PC2: 16.1%; PC3: 13.4%). The variables most positively associated with the first axis (PC1) were most of the frequency variables (start, median, lower, end, peak, upper, bandwidth; component loadings: 0.67–0.87), along with entropy (component loading: 0.67), while the variable most negatively associated was duration (component loading: − 0.68). Therefore, higher PC1 values refer to calls of higher frequency and shorter duration, while the opposite is the case for lower values. Range was the variable most negatively associated with PC2 (component loading: − 0.87), while bandwidth and entropy were most positively associated (component loadings: 0.65 and 0.51). As such, higher PC2 values refer to calls with a larger range in frequency and higher entropy. Three variables were positively associated with PC3 (amplitude modulation, frequency modulation, and number of inflection points; component loadings: 0.70–0.73), thus higher PC3 values refer to calls with more variation in frequency and amplitude. For all calls except droplets, the HI calls showed a greater degree of within-call type variation in both PC1 and PC2, but the variation overlapped between populations within each AV classified call type (Figs. 3, S1). The NL calls tended to be lower frequency and longer duration, relative to HI calls (i.e. lower PC1 values), with the exception of droplets (Figs. 3, S1). The NL swops, teepees, and whups tended to have lower frequency ranges and entropy (i.e. lower PC2 values) relative to HI calls of the same type but higher frequency ranges and entropy for droplets and growls in NL relative to HI (Figs. 3, S1). All calls in NL tended to have less variation in amplitude and frequency (i.e. lower PC3 values) relative to HI (Fig. 3).

Classification and regression tree (CART) and random forest (RF) analyses had an overall agreement with AV classification of 83% (n = 1531/1841) and 77% (n = 1416/1841), respectively (Table 3). In the RF, the most important splitting variables were end frequency, frequency trend, duration, start frequency, and upper frequency. In both the CART and RF, droplets, growls, and swops had individual agreements > 85%, while the teepees and whups had 40–65% agreements (Table 3). When misclassified, whups were primarily misclassified as growls (87–94% of those misclassified, n = 104/119 (CART), 181/193 (RF)) and growls were primarily misclassified as whups (94–95% of those misclassified, n = 51/54 (CART), 63/66 (RF)). Teepees were primarily misclassified as swops (56–62% of those misclassified, n = 39/70 (CART), 54/87 (RF)).

**Table 3:** CART and RF confusion matrices with sample size (n) for each call type, showing the number of cases correctly assigned to each observer classified call type in bold along the diagonal.

	n	Droplet	Growl	Swop	Teepee	Whup	Agreement
**CART**							
Droplet	341	**313**	5	19	2	2	92
Growl	515	0	**461**	0	3	51	90
Swop	476	24	0	**437**	15	0	92
Teepee	180	7	10	39	**110**	14	61
Whup	329	0	104	2	13	**210**	64
**RF**							
Droplet	341	**300**	0	40	1	0	88
Growl	515	0	**449**	0	3	63	87
Swop	476	23	0	**438**	15	0	92
Teepee	180	5	10	54	**93**	18	52
Whup	329	0	181	1	11	**136**	41

Percent classification success in the CART and RF is given for each call type in the last column. The CART had an overall classification success of 83% (n = 1531/1841) and the RF 77% (n = 1416/1841). In the RF, the most important variables based on the Gini index were end, trend, dur, start, and upper. Variable abbreviations correspond to those in Table 4.

## Discussion

Our study quantitatively demonstrates that at least five call types are present within the humpback whale repertoire across genetically independent populations, generational time, and contrasting behavioral contexts. We found that humpback whales on Hawaiian (HI) breeding grounds in the 1980’s used the same call types as foraging humpback whales in Newfoundland (NL) in the 2010’s. These populations have not shared a common habitat in several million years^41^, and exchange among ocean basins (North Atlanic, North Pacific, and Southern Oceans) is rare^39–41^, making it highly unlikely that these five call types persisted as a result of cultural transmission among these two populations. However, we cannot rule out that the calls are maintained via cultural transmission among whales within each ocean basin (North Atlantic and North Pacific) or via vertical cultural transmission from mother to calf through vocal learning. Despite the contrast in behavioral context between the two regions (foraging versus reproduction), pieces of the humpback whale call repertoire were shared and persisted across multiple generations, demonstrating the behavioral ubiquity of these calls and indicating that they are likely fixed. The focal call types investigated in this study are also structurally analogous to call types recorded on two migration routes^56,61,62^ and two foraging grounds spanning the 1970’s to the 2010’s^25,54,57^. Overall, our findings, together with these other studies, provide support that these call types may be common to all humpback whale populations.

Given the widespread use and presence of these call types, it is likely—as has been suggested elsewhere^25,55,68^—that these calls are important for mediation of social interactions^61,73,74^, such as maintaining contact (e.g., between mother and calf) or in other close range communication^55,68,69^. Given that these proposed functions are likely to be ubiquitous and important to all humpback populations, regardless of context, we would expect these calls to persist over time. In support, stable calls in various cetaceans have been suggested to be important for maintaining individual associations including group coordination^28,61,75^, mother–offspring contact^55,67^, and individual recognition^15^. Alternately, the universality of certain call types may suggest their versatility, depending on immediate context. For instance, a single call type may not necessarily convey a single message; instead it might convey different messages depending on the motivational state of the producer^67,76^, serve as a contact call, or indicate one meaning in the context of a feeding group of whales and something different when used in inter-individual aggressive situations.

Though the PCA indicated that there is variability in call type acoustic parameters between populations (Figs. 3, S1), the often high agreement between AV classification and the CART and RF, confirmed that calls from the two populations shared enough acoustic properties to be robustly grouped into call types. This between-population variation within calls in acoustic characteristics, particularly frequency and duration, may reflect differences in the recording environments, including different oceanographic conditions, bottom substrates, wave action and proximity to vocalizing whales which may have led to divergent propagation effects of calls between the two regions^77,78^. For instance, although the same signal-to-noise ratio was used in both datasets, recordings in HI were made while whales were within ~ 200 m of the hydrophone and made in the presence of singing whales^46^, while whales were likely at a variety of distances from the hydrophone in NL and calls were not obscured by background singers. While the technical specifications of the recordings were adjusted to maximize comparability, the behavior of the whales and the recording conditions were beyond our logistical control.

The between-population variation in acoustic characteristics could also be due to differences in social and behavioural contexts, which may relate to motivation-structural rules^67,76^. The HI recordings were mainly from surface-active, aggressive males^46^, presumably competing for mates^43,46–48^. In contrast, NL whales were feeding on highly abundant aggregations of their main prey, capelin (*Mallotus villosus*^79^), and aggressive behaviour was not observed among whales. Calls in NL tended to be lower frequency and longer duration (exception: droplets), with smaller frequency ranges and less entropy and amplitude variation (exceptions: droplets and growls) relative to calls in HI (Figs. 3, S1). The larger frequency ranges in HI calls and, to some extent, entropy and amplitude variation, could reflect the agressive context, as broader bandwidths and higher variability are often associated with these^67,76^, while the higher frequencies might reflect fear or distress^67,76^. As noted previously, frequency and duration differences may be related more to the recording conditions, whereby the close proximity of hydrophones to vocalizing whales in HI may have minimized propagation loss of higher frequency components of calls relative to NL. The differences in behavioural context, however, may explain why swops and teepees in HI occurred more often in bouts and in longer bouts than their NL counterparts (Table 2). In support, vocalization rates in Hawaii (breeding) increased during joining events and, calling is known to be more frequent in groups of three or more than in other social contexts^46^; call bouts were most common during joining events along the eastern coast of Australia (migration)^80^, and overall vocalization rates were highest for lone males when leaving a group^55^. Divergent proportional call use between populations might also be related to context, but behavioural studies to understand the function of these calls will be necessary for further interpretation.

The presence of a shared call repertoire by both populations was supported by both analysis types in this study, but lower levels of agreement were apparent for some call types, consistent with earlier humpback whale call classification studies^25,54,56,62^. In particular, whups and growls were commonly misclassified as one another. These call types share many acoustic features, and are generally discriminated by the presence of an upsweep at the end of the whup, which is absent in growls. Although some of the misclassifications may be due to between-population variation within call types, the low agreement for whups and growls has been found in other studies^25,54,57,81^ and we, therefore, posit that these two call types may be one type, influenced either by behavioural or individual variation. In Alaska, whups have been proposed to function to maintain contact between individuals, as they share general acoustic characteristics with a call used by right whales (*Eubalaena glacialis*) for this function and appeared to occur in bouts, in one region, that may indicate counter-calling among individuals^68^. Growls also appear to be used during social interactions of humpback whales, particularly in contexts of higher arousal, such as groups of three or more adults when males compete for the position of escort of a female^67^. Further devoted study of the function of whups and growls will be necessary to determine whether separating them is biologically relevant, or if they occur in similar behaviour contexts and should be grouped together^56^.

In conclusion, we have provided evidence of the presence of the same humpback whale call types on allopatric foraging and breeding grounds with decadal time-scale separation. Long-term studies of call stability and use in more regions over multiple generations will further elucidate whether these and other humpback whale call types are stable in additional regions and fixed across generations, and provide further evidence that these calls, and possibly others, form the foundation of the repertoire. The finding of persistence across generational time, ecological context, and genetic and geographic distance could suggest a genetic component in the maintainence of these call types^24,25^, however, they could also be maintained through cultural transmission.

Regardless of how these calls are maintained in the repertoire, their persistence suggests their importance and provides support for their universality. Thus, these calls may provide reliable indicators of humpback whale presence and represent useful diagnostics to the species level in passive acoustic monitoring studies. Acoustic monitoring has become a powerful tool in detecting the presence of vocalizing marine mammals in numerous contexts (e.g., studies of distribution or migration) and for monitoring marine mammals in the vicinity of human activities (e.g., shipping, military operations^82,83^). However, the technology relies not only on animals vocalizing to enable a detection, but also on reliably identifying the species based on vocalization characteristics (i.e., based on differences in acoustic structures), highlighting the importance of persistent and widespread calls.

## Methods

Underwater recordings of humpback whale vocalizations, using a moored hydrophone, were made on a foraging ground on the northeastern coast of Newfoundland (NL), Canada (Fig. 1) during July–August 2015 and 2016. Boat-based recordings were made in waters off west Maui, Hawaii (HI), USA (Fig. 1) during January-April 1981 and February-April 1982 using a portable hydrophone when whales were within ~ 200 m or less^46^ (see Table 1 for details). During recordings in HI, concurrent behavioural observations were made of the focal animals^46^. In NL, concurrent behavioural data were not collected, but anecdotal observations were made during photo-identification studies, including the timing of arrival of humpback whales within < 5 km from the hydrophone^79^. For each year, recordings were analyzed starting from the first day humpback whales arrived within < 5 km of the hydrophone until whales were no longer observed and/or no calls were found for 48 h on recordings (July 15–22, 2015; July 29–August 8, 2016).

Recordings from both regions were reviewed in *Raven Pro* 1.5 or 2.0 (hereafter referred to as *Raven*^84^) using a Hann window, 8192 (NL) and 32,768 (HI) Discrete Fourier Transform, 2.93 Hz resolution, and 50% overlap. Spectrograms and descriptions from the literature^25,54,57^, as well as examples from exisiting datasets, were used for comparison to identify the five call types of interest (swops, droplets, teepees, whups, growls) in the recordings using aural/visual (AV) characteristics. All calls that were deemed through AV classification to be one of the five call types of interest were annotated in time and frequency by one or two trained observers (MVE & MEHF) and were labelled with the call type name. To be included in this study, a call had to have a clearly distinguishable start and end time and could not be overlapping with other biological or non-biological sounds. Acoustic features were measured using either *Raven* or the Noise-Resistant Feature Set (NRFS)^85^ (Table 4). The NRFS was used as it is considered to be robust to variation in noise conditions and in user annotated selection boxes^54,57,85^. Only calls with an SNR between 10 and 25 dB above ambient noise were retained for analysis^54,56,61^; this range was chosen to ensure comparable, high quality calls from both regions in the final dataset. Calls meeting these inclusion criteria were randomized and re-classified to ensure correct assignment to one of the five types. Sixteen variables were measured from call features, mainly using the NRSF (see definitions in Table 4), with the exception of start and end frequency and bout, that were measured manually in *Raven*^54,61,86^. All frequency variables were log-transformed prior to analysis^54,56^.

**Table 4:** Descriptions of the variables that were used in the CART and RF.

Variable name	Unit	Abbreviation	Description
Lower frequency*	Hz	Lower	Lowest frequency of the call
Upper frequency*	Hz	Upper	Highest frequency of the call
Frequency range	Hz	Range	Ratio of lower to upper frequency
Duration*	s	Dur	Length of the feature box
Bandwidth*	Hz	Band	Height of the feature box
Median frequency*	Hz	Median	The frequency at which 50% of the energy is to either side
Frequency of peak overall intensity*	Hz	Peak	The frequency with the greatest energy/amplitude in the feature box
Amplitude modulation rate*	Rate	Ampmod	Dominant rate of amplitude modulation
Frequency modulation rate*	Rate	Freqmod	Dominant rate of frequency modulation
Overall entropy*	Bits	Entropy	Measure of how evenly energy is distributed across the frequencies
Upsweep fraction*	%	Upsweep	Fraction of time that the median frequency in one time block is greater than the preceding time block
Bout		Bout	Number of the same call type in sequence in a discrete period of time (< 2 s separation between instances)
Start frequency	Hz	Start	Frequency at the beginning of the call measured on the fundamental frequency or lowest harmonic
End frequency	Hz	End	Frequency at the end of the call measured on the fundamental frequency or lowest harmonic
Frequency trend	Hz	Trend	Ratio of start to end frequency
Number of inflection points in the peak frequency contour		Inflection	Count of the number of times the slope of the peak frequency contour changes

Variables marked with a * were measured using the Noise-Resistance Feature Set (the same descriptions as for the program *Osprey* from Mellinger and Bradbury (2007)). The Noise-Resistant Feature Set placed a smaller feature box within the manually created selection box in *Raven* 1.5 and 2.0. Measurements of the call were made based on the contents of the feature box. Start frequency, end frequency and bout were determined manually in *Raven*. All frequency variables (i.e. with unit Hz) were log-transformed. Units are provided where applicable. Abbreviations are given for each variable that correspond to the other tables and figures.

To examine between-population variation within each AV classified call type, we performed a Principal Component Analysis (PCA) to reduce all 16 acoustic variables into a smaller number of variables that explained most of the variation in the dataset and then examined boxplots of these newly derived variables (i.e. principal components). We used this descriptive method as our data violated the underlying assumption of independence for parametric and non-parametric statistics. To assess whether between-population variation in acoustic characteristics within call types influenced classification into AV classified call types, all 16 variables were also included in CART and RF analyses, which were conducted using the *rpart* and *randomforest* packages in *R* (version 3.5.0). Both analyses were run with all five AV classified call types. The Gini index was used in the CART analysis to determine the “goodness of split” at each node^87^ and terminal nodes were set to a minimum size of 10 samples. In the RF, the number of predictors considered at each node was set to three, the Gini index was used to assess their importance, and 1000 trees were grown^57,61^. For both tests, classification agreement between AV classification and classification based on acoustic measurements was assessed, whereby high agreement (≥ 70%) provided further evidence that the acoustic characteristics showed less variation within an AV-classified call type than among call types, despite between-population variation among call types.

## Supplementary Information

Supplementary Figure S1.

## Data Availability

The datasets generated during and/or analysed during the current study are available from the corresponding author on reasonable request.
