# Supplementary material for: Allopatric humpback whales of differing generations share call types between foraging and wintering grounds
Source: Sci Rep. 2021 Aug 11;11:16297. doi: 10.1038/s41598-021-95601-7 (PMC8357822; doi:10.1038/s41598-021-95601-7)
Supplement: Supplementary file 1 — Supplementary Figure S1. [file 41598_2021_95601_MOESM1_ESM.pdf]

(A) All Calls

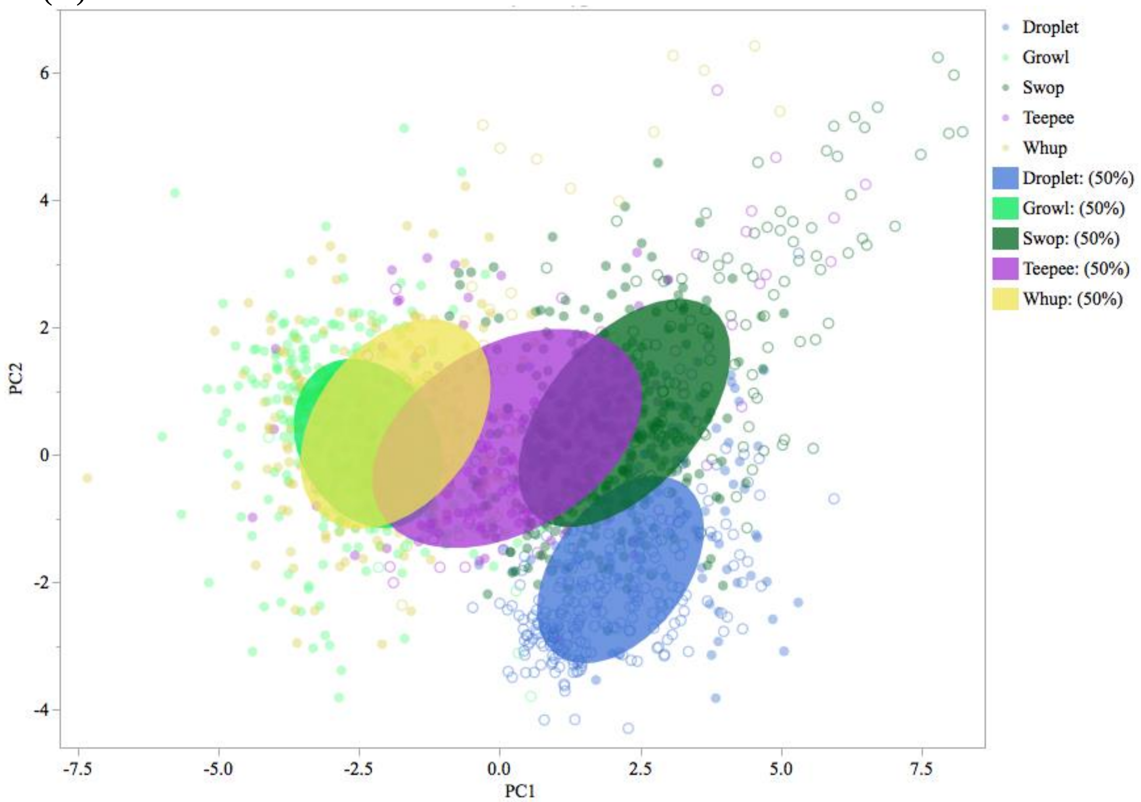

(B) Whup

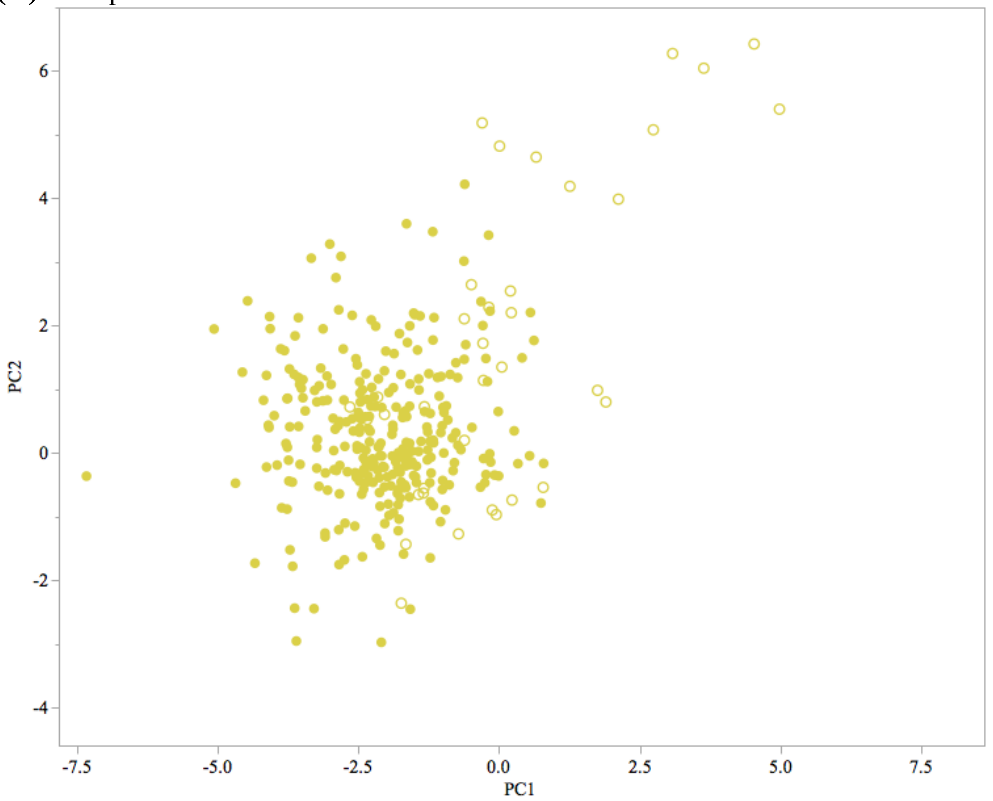

(C) Growl

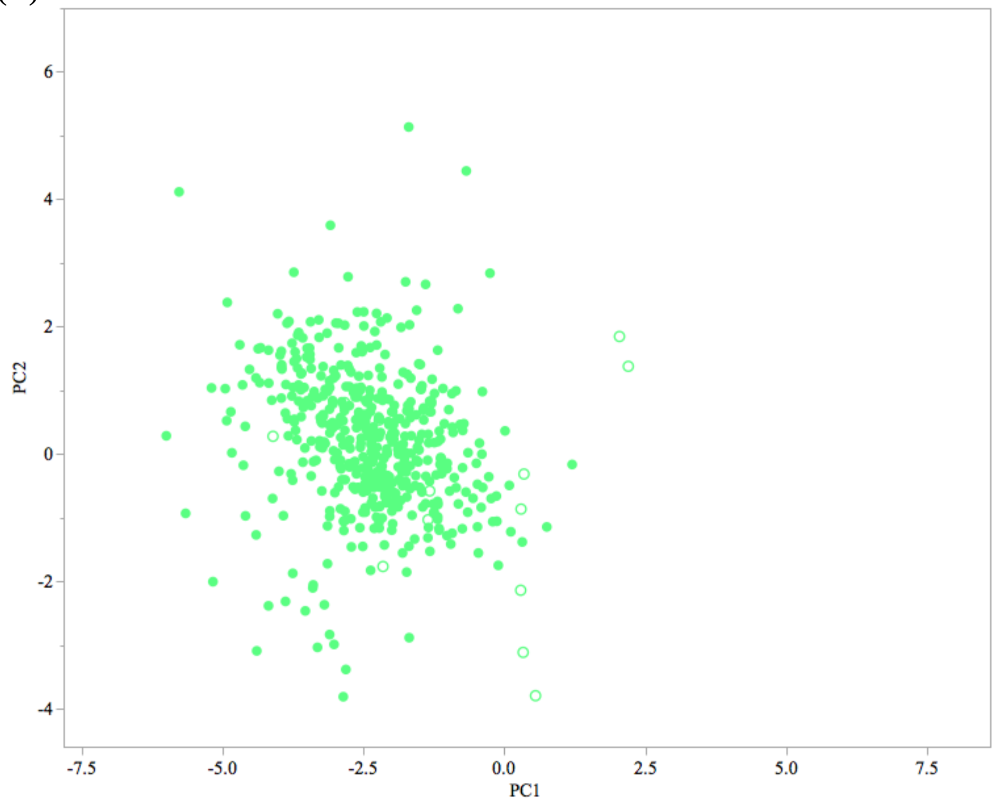

(D) Swop

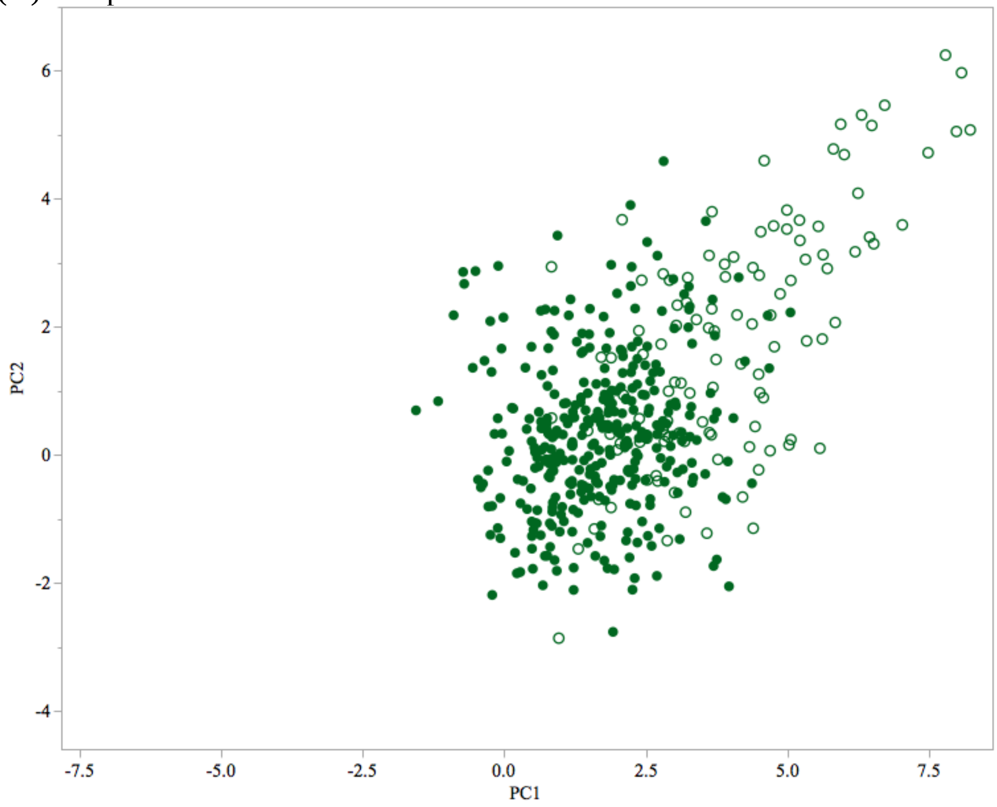

(E) Droplet

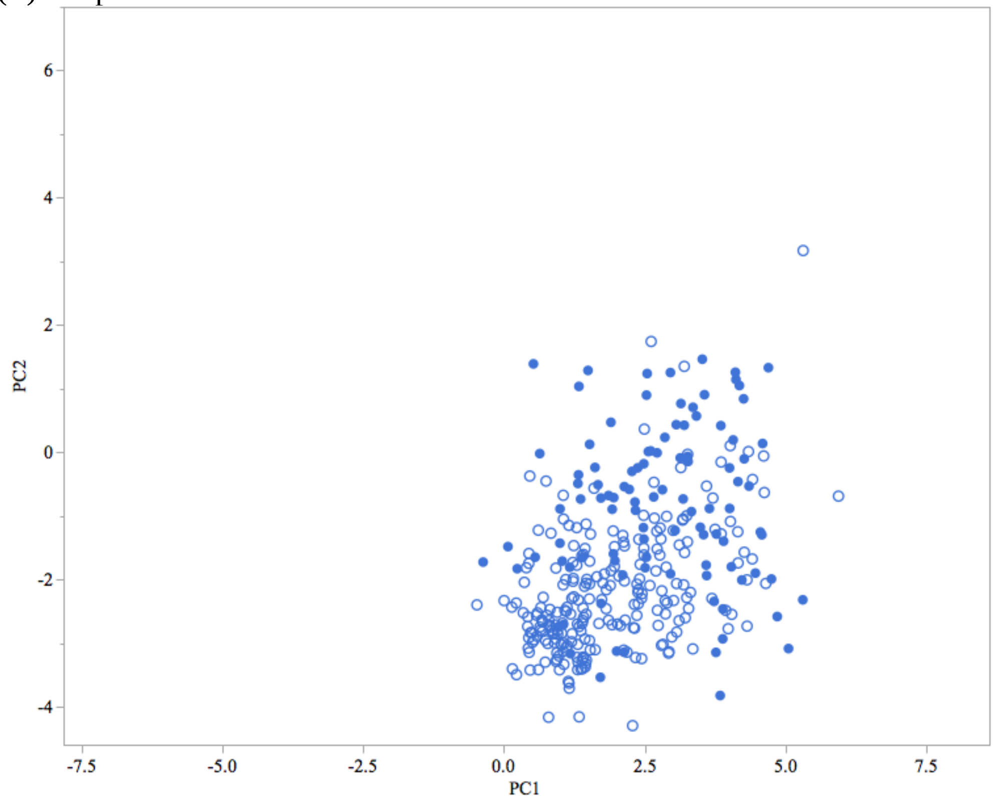

(F) Teepee

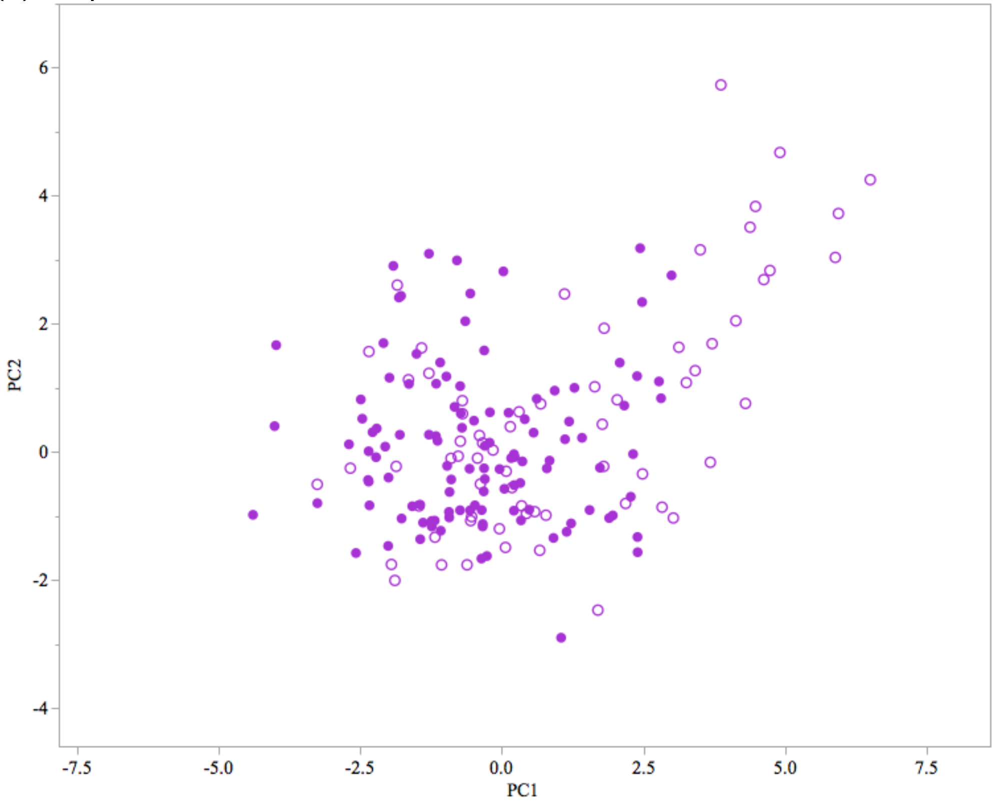

Figure S1. Biplots of PC1 versus PC2 showing between-population variation in acoustic parameters within call types as well as variation among call types. The individual cases of each of the call types from Newfoundland are shown as solid circles and those from Hawaii as hollow circles. The 50% ellipse area is also shown in (A) for all call types. (A) Shows all call types together to exemplify the variation among the call types, (B-F) show each call type individually on the same scale to exemplify the variation within each call type.
